# 

**Published:** 1891-05

**Authors:** 


### CONTENTS.

COMMUNICATIONS.

Scientific Investigations of the Cranium and Jaws........................ 213
Phagocytosis in Dental Lesions........................................ 220
Why Copper Amalgam sometimes wastes in the Mouth.................. 226
The relations of Bacteria to Practical Surgery.......................... 235
An Eye to Business.................................................... 246

PROCEEDINGS.

The Forty-Seventh Annual Meeting of the Mississippi Valley Association of Dental Surgeons........................................ 247
Peroxide of Hydrogen.................................................. 250

COMMENCEMENTS.

American College of Dental Surgery, Chicago............................ 251
Ohio College of Dental Surgery........................................ 251
New York College of Dentistry........................................ 252
Vanberbilt University—Department of Dentistry......................... 253
Philadelphia Dental College........................................... 254
Dental Department of the Northwestern University...................... 255
The Columbian University, Dental Department, Washington, D. C. 255
Pennsylvania College of Dental Surgery................................. 256
University of Iowa, Dental Department.................................. 257

EDITORIAL.

Mistakes of Physicians................................................ 258
Parts and Percentage.................................................. 260
Dental Law............................................................ 262
A Pleasant Visit...................................................... 263
An Important Announcement............................................. 264

